# GPS-SNO: Computational Prediction of Protein *S*-Nitrosylation Sites with a Modified GPS Algorithm

**DOI:** 10.1371/journal.pone.0011290

**Published:** 2010-06-24

**Authors:** Yu Xue, Zexian Liu, Xinjiao Gao, Changjiang Jin, Longping Wen, Xuebiao Yao, Jian Ren

**Affiliations:** 1 Hubei Bioinformatics and Molecular Imaging Key Laboratory, Department of Systems Biology, College of Life Science and Technology, Huazhong University of Science and Technology, Wuhan, Hubei, China; 2 Hefei National Laboratory for Physical Sciences at Microscale and School of Life Sciences, University of Science and Technology of China, Hefei, China; 3 Life Sciences School, Sun Yat-sen University (SYSU), Guangzhou, Guangdong, China; King Abddulah University of Science and Technology, Saudi Arabia

## Abstract

As one of the most important and ubiquitous post-translational modifications (PTMs) of proteins, *S*-nitrosylation plays important roles in a variety of biological processes, including the regulation of cellular dynamics and plasticity. Identification of *S*-nitrosylated substrates with their exact sites is crucial for understanding the molecular mechanisms of *S*-nitrosylation. In contrast with labor-intensive and time-consuming experimental approaches, prediction of *S*-nitrosylation sites using computational methods could provide convenience and increased speed. In this work, we developed a novel software of GPS-SNO 1.0 for the prediction of *S*-nitrosylation sites. We greatly improved our previously developed algorithm and released the GPS 3.0 algorithm for GPS-SNO. By comparison, the prediction performance of GPS 3.0 algorithm was better than other methods, with an accuracy of 75.80%, a sensitivity of 53.57% and a specificity of 80.14%. As an application of GPS-SNO 1.0, we predicted putative *S*-nitrosylation sites for hundreds of potentially *S*-nitrosylated substrates for which the exact *S*-nitrosylation sites had not been experimentally determined. In this regard, GPS-SNO 1.0 should prove to be a useful tool for experimentalists. The online service and local packages of GPS-SNO were implemented in JAVA and are freely available at: http://sno.biocuckoo.org/.

## Introduction

**Figure 1:**
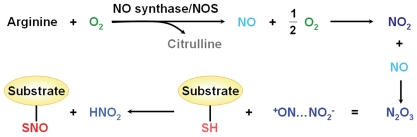
The biochemical processes of the endogenous NO source and protein *S*-nitrosylation.

The 1998 Nobel Prize for Physiology or Medicine was awarded for seminal discoveries that showed nitric oxide (NO) to be a freely-diffusible signaling molecule and second messenger which regulates the production of cyclic GMP (cGMP) and plays essential roles in the cardiovascular system. Subsequently, a large number of studies challenged this fundamental view by demonstrating that NO could spatially and temporally target specific cysteine thiols and transition metals of proteins, a reversible post-translational modification (PTM) termed *S*-nitrosylation [1]–[6]. In most cell types, NO synthases (NOSs) catalyze the reaction of arginine and O_2_ to produce citrulline and endogenous NO (Figure 1). NO can then be further oxidated into NO_2_ and processed into N_2_O_3_ (Figure 1). By direct interactions or through scaffold and adaptor proteins, protein targets closely associated with NOS may be *S*-nitrosylated *in situ* to form *S*-nitrosothiols (SNOs) (Figure 1) [1]–[4]. Although the enzymatic mechanisms of protein *S*-nitrosylation are still elusive, several enzymes have been demonstrated to facilitate *S*-nitrosylation or de-nitrosylation reactions. For example, Cu, Zn superoxide dismutase (SOD) and thioredoxin (TRX) promote *S*-nitrosylation, while protein disulfide isomerase (PDI) is suggested to regulate de-nitrosylation [3], [4]. Recent reports have proposed that *S*-nitrosylation can modulate protein stability [7], activities [8] and trafficking [9], [10], and play an important role in a variety of biological processes, including transcriptional regulation [7], cell signaling [11], apoptosis [8], and chromatin remodeling [12]. Moreover, aberrant *S*-nitrosylation has been implicated in numerous diseases and cancers [1], [2], [8]. In this regard, experimental identification of *S*-nitrosylated proteins together with their sites would serve as a foundation of understanding the molecular mechanisms and regulatory roles of *S*-nitrosylation.

Conventional experimental identification of *S*-nitrosylation sites with a site-directed mutagenesis strategy is laborious and of low-throughput [7], [8]. In 2001, Jaffrey *et al.* developed a novel biotin switch technique (BST) for the large-scale detection of cellular *S*-nitrosylated substrates [13], [14]. The BST comprises three steps, including methylthiolation of free cysteine thiols with methyl methanethiosulfonate (MMTS), reduction of SNOs to thiols with ascorbate, and ligation of the nascent thiols with *N*-[6-(biotinamido)hexyl]-3′-(2′-pyridyldithio)-propionamide (biotin-HPDP) [5], [13], [14]. Together with state-of-the-art mass spectrometry (MS), BST was successfully used to discover a large number of potential *S*-nitrosylated proteins in *H. Sapiens* [15], [16], *M. musculus* [17], and *A. thaliana* [18]. Recently, several approaches, including SNOSID (SNO-Cys site identification) [19], [20], were also developed to determine potential S-nitrosylation sites from MS-derived data [19]–[23].

Currently, computational studies of post-translational modifications (PTMs) are attracting considerable attention. In contrast with time-consuming and expensive experimental methods, certain of the accurate and convenient computational approaches have been shown to be able to rapidly generate helpful information for further experimental verification. Although there have been ∼170 databases and computational tools developed for PTM analyses (http://www.biocuckoo.org/link.php), *in silico* prediction of S-nitrosylation sites in proteins is still a great challenge. In a previous study, Hao *et al.* tested the prediction performance using a training data set containing 65 positive *S*-nitrosylation sites and 65 negative samples [20]. The support vector machines (SVMs) algorithm was used, and the performance result was disappointing [20].

In this work, 504 experimentally verified *S*-nitrosylation sites in 327 unique proteins were obtained from the scientific literature and public databases (Supplementary Table S1). Previously, we developed the algorithm GPS 2.0 (“Group-based Prediction System”) for the prediction of kinase-specific phosphorylation sites [24]. Here, we report substantial improvement of the method and the release of the GPS 3.0 algorithm. Then we developed a novel computational software of GPS-SNO 1.0 for prediction of *S*-nitrosylation sites. The leave-one-out validation and 4-, 6-, 8- and 10-fold cross-validations were calculated to evaluate the prediction performance and system robustness. By comparison, the performance of the GPS 3.0 algorithm was better than several other approaches, with an accuracy of 75.80%, a sensitivity of 53.57% and a specificity of 80.14% under the low threshold condition. As applications of GPS-SNO 1.0, we also collected 485 potentially *S*-nitrosylated substrates from PubMed (Supplementary Table S2). These proteins were detected from large-scale or small-scale studies, and the exact *S*-nitrosylation sites had not been experimentally determined. We predicted 359 (∼74%) of these targets with at least one potential *S*-nitrosylation site. These prediction results might be of use for further experimental verification. Finally, the online service and local packages of GPS-SNO 1.0 were implemented in JAVA 1.4.2 and are freely available at: http://sno.biocuckoo.org/.

## Methods

### Data preparation

We searched the scientific literature from PubMed with the keywords of “nitrosylation” or “nitrosylated”, and collected 549 experimentally verified *S*-nitrosylation sites in 363 proteins which were published before Jun. 23^rd^, 2009. We also searched the sequence annotations of the UniProt database (http://www.uniprot.org/uniprot/) [25]. Only experimentally verified *S*-nitrosocysteine sites were reserved. Potentially nitrosylated sites with annotations of “By similarity”, “Potential” or “Probable” were removed. From the UniProt database, in total we obtained 22 known *S*-nitrosylation sites in 18 proteins. In a previous study, Li *et al.* developed the public database SysPTM and collected 50 PTM types with experimentally verified information [26], while the known *S*-nitrosylation sites were taken from two large-scale surveys [20], [27]. The three data sets were integrated, while the protein sequences were retrieved from the UniProt database.

As previously described [24], [28]–[31], we regarded the cysteine (C) residues that undergo *S*-nitrosylation modification as positive data (+), while all other non-nitrosylated cysteines were taken as negative data (−). The positive data (+) set for training contain a number of homologous sites from homologous proteins. If the training data were highly redundant with too many homologous sites, the prediction accuracy would be overestimated. To avoid such overfitting, we clustered the protein sequences with a threshold of 40% identity by CD-HIT [32]. If two proteins were similar with ≥40% identity, we re-aligned the proteins with BL2SEQ, a program in the BLAST package [33], and checked the results manually. If two *S*-nitrosylation sites from two homologous proteins were at the same position after sequence alignment, only one item was reserved while the other was discarded. Finally, the non-redundant data set for training contained 504 positive sites and 2,581 negative sites from 327 unique substrates. The 504 experimentally verified *S*-nitrosylation sites are presented in Supplementary Table S1.

### Performance evaluation

As previously described [24], [28]–[31], we used four measurements, including sensitivity (*Sn*), specificity (*Sp*), accuracy (*Ac*), and Mathew Correlation Coefficient (*MCC*) to evaluate the prediction performance of GPS-SNO 1.0. The four measurements were defined as below:
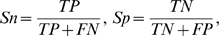


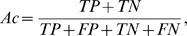
and 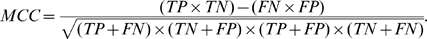.

In this work, the leave-one-out validation and 4-, 6-, 8-, 10-fold cross-validations were performed. The Receiver Operating Characteristic (ROC) curves and AROCs (area under ROCs) were also carried out.

### The GPS 3.0 algorithm

For prediction of the *S*-nitrosylation sites, we greatly refined our previously developed method and released GPS 3.0 (Group-based Prediction System) algorithm, with its two major components of scoring strategy and performance improvement.

The basic hypothesis of the scoring strategy is that similar short peptides might bear similar 3D structures and biochemical properties [24], [28]–[31]. First, we defined a *nitrosylation site peptide* NSP(*m*, *n*) as a cysteine (C) amino acid flanked by *m* residues upstream and *n* residues downstream. Then we used an amino acid substitution matrix, e.g., BLOSUM62, to calculate the similarity between the two NSP(*m*, *n*) peptides. For two amino acids *a* and *b*, let the substitution score between them in the amino acid substitution matrix be *Score*(*a*, *b*). Then the substitution score between the two NSP(*m*, *n*) peptides *A* and *B* was defined as:
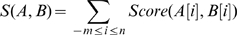
If *S*(*A*, *B*)<0, we simply redefined it as *S*(*A*, *B*) = 0.

The performance improvement process is comprised of four sequential steps of *k*-means clustering, peptide selection (PS), weight training (WT) and matrix mutation (MaM).

#### 1) *k*-means clustering

The *k*-means clustering method has been extensively used in analyses of gene [34], [35] or protein [36] expression data, protein 3D structural analysis [37], and image processing [38], [39]. Here, we used this approach to cluster the training data set into several groups. In these studies, more clusters will generate better performance. However, the current training process is excessively time-consuming. Therefore, to improve the calculation speed, the K was roughly set to 3. Given two NSP(*m*, *n*) peptides *A* and *B*, the similarity was measured as:
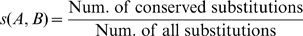
A conserved substitution is a substitution with a *Score*(*a*, *b*)>0 in the BLOSUM62 matrix. The *s*(*A*, *B*) ranges from 0 to 1. Thus, the distance between them can be defined as: *D*(*A*, *B*) = 1/*s*(*A*, *B*). If *s*(*A*, *B*) = 0, *D*(*A*, *B*) = ∞.

By exhaustive testing, NSP(7, 7) was used for this procedure. First, three *S*-nitrosylation sites from the positive data (+) were randomly chosen as the centroids. Second, the other positive sites were compared in a pairwise manner with the three centroids and clustered into groups with the highest similarity values. Third, the centroid of each cluster was updated with the highest average similarity (HAS). The second and third steps were iteratively repeated until the clusters did not change any longer. After the three clusters for the positive sites had been determined, we put each negative site into the cluster with the HAS.

#### 2) Peptide selection (PS)

In this step, We determined the optimized combination of NSP(*m*, *n*) for optimal performance. The combinations of NSP(*m*, *n*) (*m* = 1, …, 30; *n* = 1, …, 30) were extensively tested. The optimal NSP(*m*, *n*) for each cluster was separately selected, with the highest leave-one-out performance by singling out one site (all sites must be singled out one time). The *Sp* value was fixed at 80%.

#### 3) Weight training (WT)

The weight of each position in NSP(*m*, *n*) was initially defined as 1. The leave-one-out performance was calculated with the *Sp* of 80%. A weight of any position was randomly picked out for +1 or −1, and the leave-one-out result was re-computed. The manipulation was adopted if the *Sn* value was increased. The process was repeated until convergence was reached. Then the updated substitution score between two NSP(*m*, *n*) peptides *A* and *B* was refined as:
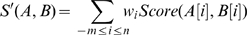
The *w_i_* is the weight of position *i*. Again, if *S′*(*A*, *B*)<0, we simply redefined it as *S′*(*A*, *B*) = 0.

#### 4) Matrix mutation (MaM)

The above three approaches were first introduced here in this work, while the MaM strategy was established in our previous work [24]. As previously described, BLOSUM62 was chosen as the initial matrix, and the leave-one-out performance was calculated. Subsequently, we fixed the *Sp* as 80% to improve the *Sn* by randomly picking out an element of the matrix for +1 or −1. The procedure was terminated when the *Sn* value was not increased any further. More detailed information of MaM is available in supplementary Text S1.

### Implementation of the online service and local packages

The online service and local packages of GPS-SNO 1.0 were implemented in JAVA and are freely available at http://sno.biocuckoo.org/. For the online service, we tested the GPS-SNO 1.0 on a variety of internet browsers, including Internet Explorer 6.0, Netscape Browser 8.1.3 and Firefox 2 under the Windows XP Operating System (OS), Mozilla Firefox 1.5 of Fedora Core 6 OS (Linux), and Safari 3.0 of Apple Mac OS X 10.4 (Tiger) and 10.5 (Leopard). For the Windows and Linux systems, the latest version of Java Runtime Environment (JRE) package (JAVA 1.4.2 or later versions) of Sun Microsystems should be pre-installed. However, for Mac OS, GPS-SNO 1.0 can be directly used without any additional packages. For convenience, we also developed local packages of GPS-SNO 1.0, which worked with the three major Operating Systems, Windows, Linux and Mac.

## Results

### Development of GPS-SNO for prediction of *S*-nitrosylation sites

Previously, we developed a novel algorithm of GPS 1.0 & 1.10 (Group-based Phosphorylation Scoring) for the prediction of kinase-specific phosphorylation sites [29], [30]. Based on the hypothesis that similar peptides possess similar biological functions, we developed a scoring strategy using an amino acid substitution matrix, BLOSUM62 [29], [30]. We also hypothesized that the *bona fide* pattern for phosphorylation modification might be compromised by the heterogeneity of multiple structural determinants with different features. Thus, to improve the prediction performance, we adopted a Markov Cluster Algorithm (MCL for short) to partition experimentally verified phosphorylation sites into several clusters [29], [30]. In GPS 2.0, we observed that different substitution matrices resulted in different levels of performance [24]. Thus, we developed a simple approach of matrix mutation (MaM), which mutated the initial matrix of BLOSUM62 into the optimal matrix having the highest leave-one-out performance [24]. The MCL method was removed in GPS 2.0 due to its poor efficiency [24]. Recently, while studying sumoylation [28] and palmitoylation [31], we classified modification sites based on either experimentally determined or putative linear motifs. However, this procedure couldn't generate satisfying performance for prediction of *S*-nitrosylation sites.

In this work, we have greatly refined the previous strategies and here release the GPS 3.0 algorithm. The scoring strategy and MaM were preserved, while three additional approaches, including *k*-means clustering, peptide selection (PS), and weight training (WT) were added. The *k*-means clustering method has been widely used in many fields [34]–[39]. Analogously, we used this method to classify the training data set into three groups, cluster A, B and C, with HAS values of 0.2475, 0.2517 and 0.2716, respectively. In our previous work, the flanking peptides were arbitrarily selected. For example, PSP(3, 3) (*phosphorylation site peptide*) was used in GPS 1.0 & 1.10 [29], [30], while PSP(7, 7) was deliberately selected in GPS 2.0 [24]. Here, we developed the PS to determine the optimal combination of NSP(*m*, *n*) based on the highest leave-one-out performance. The NSP(*m*, *n*) for cluster A, B and C were determined to be NSP(30, 7), NSP(15, 7) and NSP(8, 3). Previously, the weight of each position in a PSP(*m*, *n*) was equal to 1. Here, we developed the WT to determine the optimal weight for each position with the highest leave-one-out performance.

**Figure 2:**
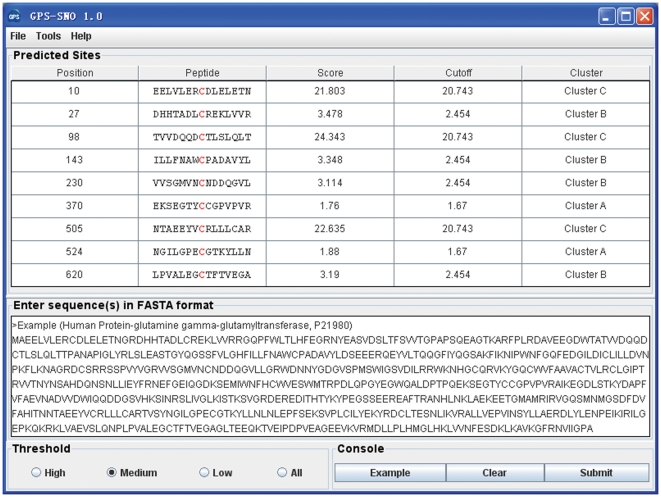
The screen snapshot of GPS-SNO 1.0 software. The medium threshold was chosen as the default threshold. As an example, the prediction results of human tissue transglutaminase (tTG, P21980) are presented.

By exhaustive testing, we decided the order of training processes to be: *k*-means clustering, PS, WT and MaM. For convenience, NSP(7, 7) is shown. The prediction results for human tissue transglutaminase (tTG, UniProt ID: P21980) are shown as an example (Figure 2). In endothelial cells, the human tTG is expressed, secreted into the extracellular matrix (ECM), and nitrosylated in a Ca^2+^-dependent manner [40]. There were fourteen unambiguous S-nitrosylation sites identified (Supplementary Table S1), including C10, C27, C98, C143, C230, C269, C277, C285, C336, C370, C371, C524, C545 and C620 [40]. The GPS-SNO 1.0 with the default threshold predicted eight sites as positive hits (Figure 2). In addition, C505 was also predicted as a positive hit, which might be shown to be useful by experimental verification.

### Performance evaluation and comparison

**Figure 3:**
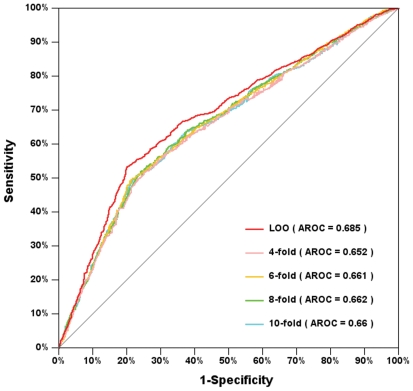
The prediction performance of GPS-SNO 1.0. The leave-one-out validation and 4-, 6-, 8-, 10-fold cross-validations were calculated. The Receiver Operating Characteristic (ROC) curves and AROCs (area under ROCs) were also carried out.

To evaluate the prediction performance and robustness of GPS-SNO 1.0, the leave-one-out validation and 4-, 6-, 8-, 10-fold cross-validations were performed. ROC curves were drawn, and the AROC values were calculated as 0.685 (leave-one-out), 0.652 (4-fold), 0.661 (6-fold), 0.662 (8-fold) and 0.660 (10-fold), respectively (Figure 3). Since the results of the 4-, 6-, 8- and 10-fold cross-validations were very similar with the leave-one-out validation, GPS-SNO 1.0 is evidently a stable and robust predictor.

**Figure 4:**
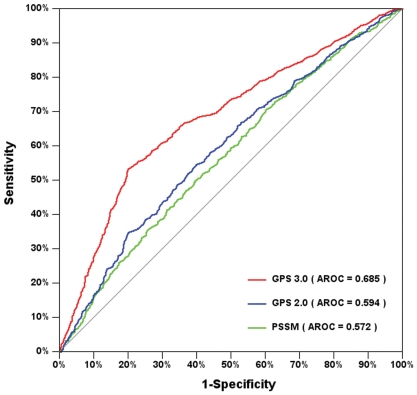
Comparison of GPS 3.0, GPS 2.0 and PSSM. For comparison, the leave-one-out results of GPS 3.0, GPS 2.0 and PSSM were calculated.

To investigate the performance of the GPS 3.0 algorithm, we compared it to several other approaches, including the GPS 2.0 and position-specific scoring matrix (PSSM) [41] methods. To avoid any bias, the same training data set used in GPS 3.0 was also employed in GPS 2.0 and PSSM. The GPS 2.0 algorithm was carried out as previously described [24], [28], [31]. For the PSSM algorithm [41], the probabilities of the twenty amino acids in terms of positive data (+) and negative data (−) were calculated as *P_+_* and *P_−_*. Then the score of a given NSP(*m*, *n*) could be calculated as:
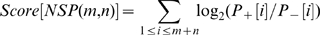



For comparison, the leave-one-out validations for the GPS 3.0, GPS 2.0 and PSSM algorithms were calculated. Again, the ROC curves were drawn, and the AROC values were calculated as 0.685 (GPS 3.0), 0.594 (GPS 2.0) and 0.572 (PSSM), separately (Figure 4). Furthermore, we fixed the *Sp* values of GPS 3.0 so as to be identical with the other methods, and then compared the *Sn* values (Table 1). For construction of the GPS-SNO 1.0 software, three thresholds of high, medium and low were established (Table 1). The results demonstrated the GPS 3.0 algorithm to be better than the other methods. In addition, previous experimental observations had suggested that *S*-nitrosylation preferred to recognize an “acid-base” motif such as K/R/H/D/E-C-/D/E [2], [3], [6]. With the training data set, we critically evaluated the performance of this motif, with an *Ac* of 82.22%, *Sn* of 4.37%, and *Sp* of 97.41%. However, with the same *Sp* value of 97.41%, the *Sn* of GPS 3.0 was 6.94% (Table 1). In this regard, the GPS 3.0 algorithm is also better than the simple motif approach.

**Table 1:** Comparison of the GPS 3.0 algorithm with other approaches.

Method	Threshold	*Ac*	*Sn*	*Sp*	*MCC*
GPS 3.0	High	80.40%	25.20%	91.17%	0.1897
	Medium	78.33%	35.32%	86.72%	0.2175
	Low	75.80%	53.57%	80.14%	0.2864
	a	82.64%	6.94%	97.41%	0.0900
GPS 2.0		78.46%	14.29%	90.98%	0.0652
		76.22%	22.22%	86.76%	0.0937
		72.66%	34.52%	80.10%	0.1299
PSSM		78.49%	13.49%	91.17%	0.0586
		75.77%	20.24%	86.60%	0.0718
		72.27%	27.58%	80.99%	0.0786
K/R/H/D/E-C-D/E^b^		82.22%	4.37%	97.41%	0.0391

For construction of the GPS-SNO 1.0 software, the three thresholds of high, medium and low were chosen. For comparison, we fixed the *Sp* values of GPS 3.0 so as to be similar or identical to the other methods and compared the *Sn* values.

*a* With the same *Sp* value, the *Sn* value of GPS 3.0 is better than the simple motif approach (6.94% vs. 4.37%).

*b* An “acid-base” motif for *S*-nitrosylation recognition [2], [3], [6].

### Large-scale prediction of *S*-nitrosylation sites in proteins

**Figure 5:**
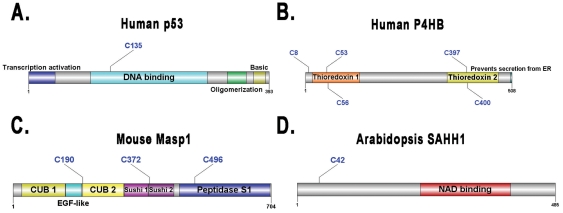
Applications of GPS-SNO 1.0. Here we predicted potential *S*-nitrosylation sites in experimentally identified *S*-nitrosylated substrates with the default threshold. (A) Human p53 (P04637); (B) Human P4HB (P07237); (C) Mouse Masp1 (P98064); (D) Arabidopsis SAHH1 (O23255).

Hundreds of proteins have been experimentally indicated to be potentially nitrosylated, with the exact *S*-nitrosylation sites in these proteins requiring elucidation. As applications of GPS-SNO 1.0, we manually collected 485 potentially *S*-nitrosylated substrates from the scientific literature (Supplementary Table S2). The primary sequences of these targets were retrieved from the UniProt database. With the default threshold (medium) of GPS-SNO 1.0, we successfully predicted 359 (∼74%) of these proteins with at least one potential *S*-nitrosylation site (Supplementary Table S2). These prediction results should be useful for further experimental verification. Several examples were randomly picked out, and their prediction results are shown in Figure 5.

It was proposed that the anticancer agent cisplatin induces *S*-nitrosylation of human p53 (UniProt ID: P04637) to prevent its translocation to mitochondria [9]. However, the *S*-nitrosylation sites in p53 were not experimentally identified. With GPS-SNO 1.0, we predicted that p53 might be nitrosylated at C135 (Figure 5A), which locates in the DNA binding region of p53, potentially influences its DNA binding affinity and regulates p53 subcellular localization (Figure 5A). As previously described [3], [4], The human protein disulfide-isomerase (PDI) P4HB (P07237) is a regulatory partner in the de-nitrosylation process. In a recent large-scale analysis [16], P4HB was also proposed as a potential nitrosylated target. Here, we predicted five potential *S*-nitrosylation sites in P4HB, including C8, C53, C56, C397 and C400 (Figure 5B). In 2003, Kuncewicz *et al.* carried out a proteomic analysis in mouse mesangial cells and identified 31 novel *S*-nitrosylated substrates [17]. We predicted one of these proteins, Mannan-binding lectin serine protease 1 (Masp1, P98064), might be nitrosylated at C190, C372 and/or C496 (Figure 5C). In addition, Arabidopsis Adenosylhomocysteinase 1 (SAHH1, O23255) was experimentally identified as a potential *S*-nitrosylated protein [18]. In this work here, we predicted that SAHH1 might only be *S*-nitrosylated at the single site of C42 (Figure 5D).

## Discussion

*S*-nitrosylation is an essential and reversible PTM of proteins [1]–[5]. Identification of *S*-nitrosylated substrates with their exact sites is fundamental for dissecting the molecular mechanisms and regulatory roles of *S*-nitrosylation [1]–[5]. In contrast with labor-intensive and expensive experimental approaches, computational prediction of *S*-nitrosylation sites is potentially a convenient and fast-speed strategy to generate useful information for subsequent experimental verification. Previously, experimental studies suggested an “acid-base” motif such as K/R/H/D/E-C-/D/E for *S*-nitrosylation recognition [2], [3], [6]. However, later researches proposed that most *S*-nitrosylation sites do not have this motif. For example, there are only ∼20 *S*-nitrosylation sites with this “acid-base” motif in our training data (Supplementary Table S1). In this regard, the simple motif approach is of only limited value.

In this report, we have greatly modified a previously developed algorithm and released the GPS 3.0 algorithm for the prediction of *S*-nitrosylation sites. All of the GPS series algorithms comprise the two major procedures of scoring strategy and performance improvement [24], [29], [30]. In GPS 1.0 & 1.10, the scoring strategy was established first, while the MCL was selected as the performance improvement step [29], [30]. In GPS 2.0, the scoring strategy was preserved, and the novel approach of matrix mutation (MaM) was used to improve performance [24]. In GPS 3.0, the original scoring strategy was adopted as the initial step. For performance enhancement, a sequential procedure was determined by means of *k*-means clustering, peptide selection (PS), and weight training (WT) and MaM. The first three approaches were newly developed in GPS 3.0. By comparison, the prediction performance of GPS 3.0 was better than other algorithms, such as GPS 2.0, PSSM and the simple motif method.

In the current stage, the data training process of GPS 3.0 was computationally intensive and time-consuming. In this regard, the technical strategies were simplified to save time. For example, in the *k*-means clustering procedure, more clusters generate better performance. However, the *k* value was set at three to improve the training speed. From our previous experience, if experimentalists want to perform a limited number of experiments to obtain at least one real site, a higher *Sp* than *Sn* value is important for avoiding too many false positive hits [24], [29], [30]. However, in some applications, experimentalists will try to exhaustively identify all the actual sites from among the predicted results without any regard to time and cost. In these cases, a higher *Sn* is more important, in order to provide more potential hits. For performance improvement, the *Sp* value was arbitrarily chosen to be 80%. Again, in the WT step, the weight of a randomly selected position was roughly added with +1 or −1. Although these parameters or settings still remain to be precisely calibrated in the future, the current GPS 3.0 algorithm has already exhibited superiority in *S*-nitrosylation site prediction. Finally, the novel software program for GPS-SNO 1.0 was implemented in JAVA.

Taken together, we propose that GPS-SNO 1.0 is a useful tool for the identification of potential *S*-nitrosylation sites. The combination of computational predictions and experimental verification will provide a foundation for an understanding of the mechanisms and the dynamics of *S*-nitrosylation.

## Supporting Information

Text S1 The algorithmic procedure of matrix mutation (MaM).
(0.07 MB DOC)

Table S1 From the scientific literature (PubMed) and the UniProt database, we collected 504 experimentally verified *S*-nitrosylation sites in 327 unique proteins. All of the sites from UniProt were complemented by the data taken from PubMed (marked in grey).
(0.07 MB XLS)

Table S2 From large-scale as well as small-scale experimental studies, we also collected 485 potentially *S*-nitrosylated substrates. The exact *S*-nitrosylation sites had not been experimentally determined. The default threshold (medium) was adopted for GPS-SNO 1.0.
(0.06 MB XLS)
