# Supplementary material for: GPS-SNO: Computational Prediction of Protein S-Nitrosylation Sites with a Modified GPS Algorithm
Source: PLoS One. 2010 Jun 24;5(6):e11290. doi: 10.1371/journal.pone.0011290 (PMC2892008; doi:10.1371/journal.pone.0011290)
Supplement: Text S1 — The algorithmic procedure of matrix mutation (MaM). (0.07 MB DOC) [file pone.0011290.s001.doc]

**The algorithmic procedure of matrix mutation (MaM)**

To improve prediction performance and robustness of the prediction system, we previously developed a novel algorithm of matrix mutation (MaM) [1]. The full technical details are shown as below:

***The following pseudocode show you how to obtain a mutated matrix：***

Initialize matrix with BLOSUM62

Set the default mutation times to 0

While mutation times less than 10000

Pick an element of matrix at random to mutate

Increase or decrease the value of the element

Calculate score of Leave-one-out with the mutated matrix

If the score increase

Keep the forward mutation

Else

Give up the mutation

Endif

Score the number of mutation times

Endwhile

Return the mutated matrix

Firstly, the amino acid substitution matrix BLOSUM62 was chosen as the initial matrix. The substitution score between two NSP(*m*, *n*) peptides *A* and *B* was defined as:

$$S(A,B)=\sum_{-m\leq i\leq n} Score(A[i],B[i])$$

If *S*(*A*, *B*)<0, we simply redefined it as *S*(*A*, *B*)=0.

And in weight training (WT) step, the updated substitution score between two NSP(*m*, *n*) peptides *A* and *B* was refined as:

$$S'(A,B)=\sum_{-m\leq i\leq n} w_{i}Score(A[i],B[i])$$

Again, if *S’*(*A*, *B*)<0, we simply redefined it as *S’*(*A*, *B*)=0.

From the definitions, the final score for a given peptide will be ≥0. Then the leave-one-out performances (*Sn* and *Sp*) were thoroughly calculated. Initially, we set the cut-off value as 0 with 0.01 increased per step, and calculated the *Sn* and *Sp* values. When the cut-off value is increased, the *Sn* value will be decreased while the *Sp* value will be enhanced. The calculation process will be stopped when the *Sn* value is decreased to be zero. In this regard, we could easily pick out the performance with *Sp* of 80%. Then we fixed the *Sp* as 80% to improve *Sn* by matrix mutation. The process of matrix mutation was stopped if the *Sn* value was not increased any more. Although matrix mutation in other types was also valid, this method could improve the leave-one-out validation significantly, which enhance the prediction performance and robustness of prediction system to data that are not included in training.

**Reference**

[1] Y. Xue, J. Ren, X. Gao, C. Jin, L. Wen, and X. Yao, GPS 2.0, a tool to predict kinase-specific phosphorylation sites in hierarchy. Mol Cell Proteomics 7 (2008) 1598-1608.
